# Multifunctional roles of leader protein of foot-and-mouth disease viruses in suppressing host antiviral responses

**DOI:** 10.1186/s13567-015-0273-1

**Published:** 2015-10-28

**Authors:** Yingqi Liu, Zixiang Zhu, Miaotao Zhang, Haixue Zheng

**Affiliations:** State Key Laboratory of Veterinary Etiological Biology, OIE/National Foot and Mouth Diseases Reference Laboratory, Key Laboratory of Animal Virology of Ministry of Agriculture, Lanzhou Veterinary Research Institute, Chinese Academy of Agricultural Sciences, Lanzhou, Gansu China; College of Veterinary Medicine, Northwest A&F University, Yangling, Shaanxi China

## Abstract

Foot-and-mouth disease virus (FMDV) leader protein (L^pro^) is a papain-like proteinase, which plays an important role in FMDV pathogenesis. L^pro^ exists as two forms, Lab and Lb, due to translation being initiated from two different start codons separated by 84 nucleotides. L^pro^ self-cleaves from the nascent viral polyprotein precursor as the first mature viral protein. In addition to its role as a viral proteinase, L^pro^ also has the ability to antagonize host antiviral effects. To promote FMDV replication, L^pro^ can suppress host antiviral responses by three different mechanisms: (1) cleavage of eukaryotic translation initiation factor 4 γ (eIF4G) to shut off host protein synthesis; (2) inhibition of host innate immune responses through restriction of interferon-α/β production; and (3) L^pro^ can also act as a deubiquitinase and catalyze deubiquitination of innate immune signaling molecules. In the light of recent functional and biochemical findings regarding L^pro^, this review introduces the basic properties of L^pro^ and the mechanisms by which it antagonizes host antiviral responses.

## Introduction

Foot-and-mouth disease (FMD) is a highly contagious disease caused by foot-and-mouth disease virus (FMDV). Outbreaks of FMD spread rapidly and usually cause devastating economic losses and trade embargoes. FMDV primarily infects cloven-hoofed animals including cattle, swine, sheep, and various ruminants. The virus belongs to the genus *Aphthovirus* in the *Picornaviridae* family and has seven serotypes: O, A, C, SAT1, SAT2, SAT3, and Asia1. There is poor cross-protection among these serotypes [1].

**Figure 1:**
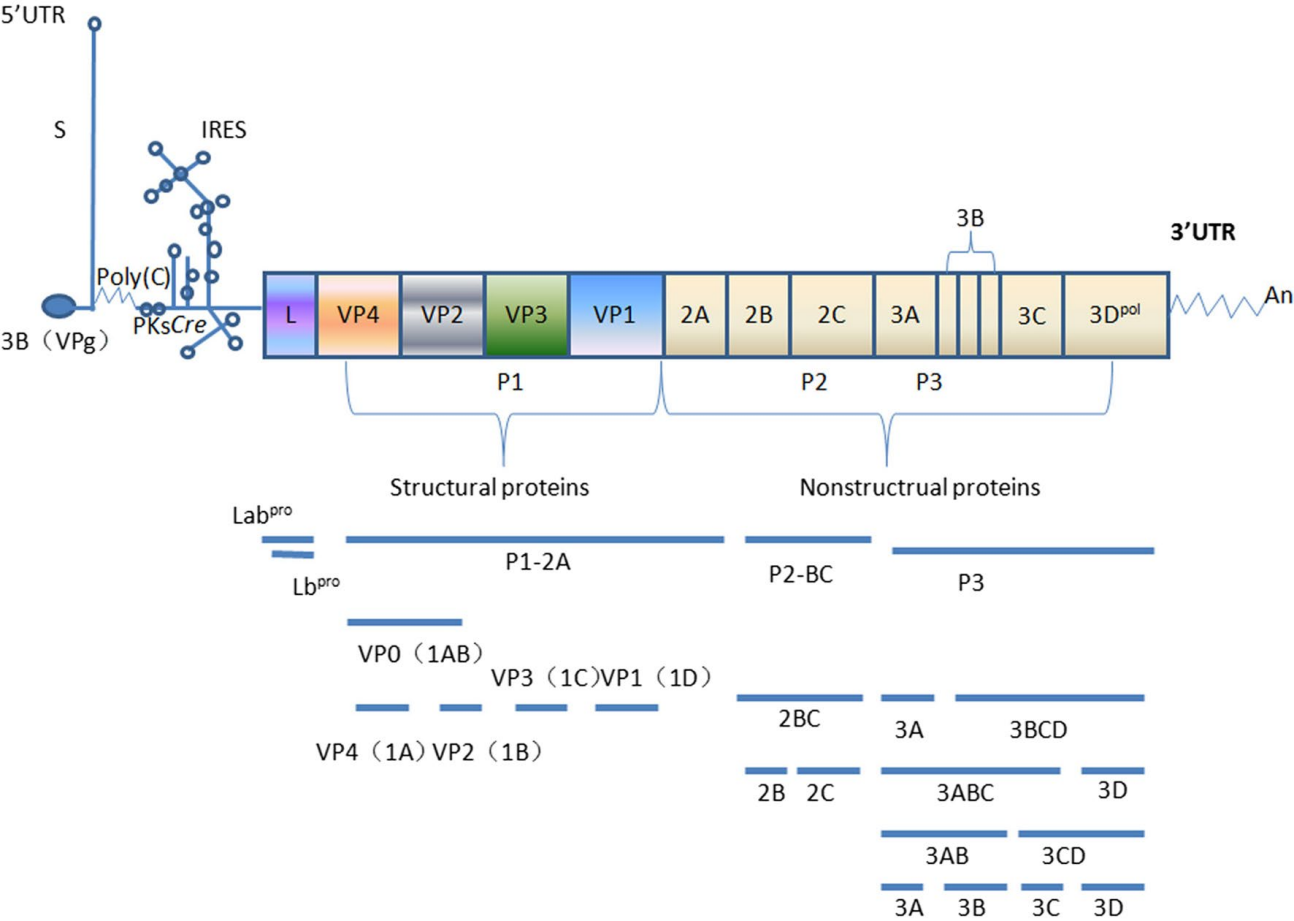
**Structure of FMDV genome and proteolytic processing of viral polyprotein.** The ORF of the viral proteins is displayed in the boxed area. The noncoding regions consist of the 5′ UTR and the 3′ UTR with a poly(A) tail. The viral functional elements in the 5′ UTR include the S fragment, the polycytidylic acid region [poly(C)], the pseudoknot structures (PKs), the *cis*-acting replicative element (*cre*), and the IRES. The 5′ end of the 5′ UTR is covalently bound to the viral 3B (or VPg) protein, which is crucial for viral RNA replication.

The genome of FMDV consists of a single-stranded positive-sense RNA with a length of about 8500 nucleotides. The genomic structure can be artificially divided into three parts: the 5′ untranslated region (UTR), the open reading frame (ORF), and 3′-UTR. The single long ORF of viral RNA encodes a polyprotein that is subsequently processed into four mature structural proteins (VP1, VP2, VP3, and VP4) which form the capsid, and about 12 non-structural proteins (L^pro^, 2A, 2B, 2C, 3A, 3B, 3C, 3D, 3AB or 3ABC, 2BC, and 3CD) (Figure 1) [2].

FMDV leader protein (L^pro^) and 3C^pro^ proteins have proteinase activity [3, 4], and are suggested to have the ability to inhibit the functions of a variety of host proteins, suppressing cellular immune responses [5–8]. For instance, 3C^pro^ and L^pro^ can induce the cleavage of host eukaryotic translation initiation factor 4 γ (eIF4G), limiting the synthesis of various host proteins [7, 9]. This could possibly include type I interferons (IFNs), indirectly promoting viral replication [10]. 3C^pro^ can also cleave the nuclear factor kappa B (NF-κB) essential modulator (NEMO) and karyopherin α1 (KPNA1) to abate innate immune signaling [5, 6]. Moreover, L^pro^ can directly cleave various other host proteins to suppress antiviral responses [11].

L^pro^, as a viral proteinase, self-cleaves from the nascent viral polyprotein precursor during FMDV infection and plays an important role in viral pathogenesis. L^pro^ has two different forms (termed Lab and Lb) due to the initiation of translation at two functional AUGs that are separated by 84 nucleotides [12]. However, the Lb AUG is more efficiently used than the Lab site despite translation initiating from the Lab site [13, 14]. Hence, Lb is more abundant than Lab. The complete loss of Lab-coding region of FMDV is reported to be lethal for the virus [15], whereas the viruses with precisely deleted Lb coding regions (leaderless viruses) were viable and could replicate both in cattle and swine. However, these viruses could not induce any pathological changes and their replicative ability was attenuated [16, 17]. Furthermore, the supernatants of primary cell cultures infected with leaderless viruses possess stronger antiviral activity than the supernatants from wild-type FMDV-infected cells [18]. Recent evidence shows that the nature and extent of the residual leader protein sequences of FMDV precisely lacking the Lb-coding sequence determine different growth characteristics in different host-cell systems [19]. Based on these studies, L^pro^ is thought to have multifunctional roles in viral pathogenicity and is considered an important virulence factor of FMDV.

L^pro^ is known to contribute to virus propagation by suppressing host antiviral activity [20]. L^pro^ has an antagonistic effect on host antiviral responses via at least three mechanisms. The most well-characterized mechanism is the cleavage of eIF4G by L^pro^, which shuts off host cap-dependent mRNA translation, and IFN translation may be included [7, 21]. Additionally, L^pro^ also directly suppresses production of IFNs (including type I and type III) at the transcriptional level, through disrupting the IFN signaling pathway to inhibit host innate immune responses [8, 22, 23]. Finally, L^pro^ can significantly inhibit the activation of some signaling transduction molecules involved in antiviral pathways through its deubiquitination enzyme (DUB) activity [22]. In this review, we discuss the current knowledge of these antagonistic mechanisms of L^pro^ against host antiviral responses.

## Different forms of FMDV L^pro^

FMDV L^pro^ shows similarities to the members of the cysteine proteinase family in structure and function [24]. It recognizes the junction sites between L^pro^ and VP4 and then cleaves itself from the polyprotein [4]. This automatic self-processing makes L^pro^ the first mature viral protein during FMDV infection. The two forms of L^pro^ (Lab and Lb) generated have been confirmed in vitro and in vivo [4, 25, 26]. Both these forms of L^pro^ exhibit the same enzymatic properties [27]. Each of them releases itself from the polyprotein via intermolecular or intramolecular self-cleavage [4, 25]. It is deemed that intramolecular self-processing is more efficient than intermolecular self-processing [28]. Nevertheless, the detailed mechanisms for the production of the two forms of L^pro^ have not been clearly elucidated. The mechanisms for selection of Lab start site (AUG1) or Lb start site (AUG2) for protein synthesis are complex. Through constructing synthetic fusion genes of AUG1 and AUG2, Belsham determined that before initiation of protein synthesis at AUG2, the ribosomes need to scan past AUG1–AUG2. The two initiation sites can both be used efficiently, whereas internal ribosome entry sites (IRESs) contribute to a slight biased utilization of the Lb site [29]. In a translation system mimicking the translation initiation pattern of the FMDV RNA observed during viral infection, the spacer region between two start codons plays a role in start codon recognition and biases the start codon selection towards the second one to initiate protein synthesis. The utilization of the first start codon depends on its sequence context [30]. Another study showed that the selection of AUG2 does not depend on the assembly of 48S complex formation on the 5′ side of AUG1 [31]. A recent study based on previous work presented by Belsham [29] revealed a mechanism involving bias-usage of translation initiation sites of L^pro^, suggesting that the poor nucleotide context of the Lab-initiation site restricts its translational efficiency. The ribosomes access the Lb site through linear scanning, starting from the upstream IRES proximal to the first initiation codon and this is not an independent entry process [14]. An early study by Poyry et al. suggested an alternative mechanism by which a few ribosomes reach the second initiation site [32].

Mutations in the initiation site of Lb disables the production of progeny viruses in transfected baby hamster kidney (BHK) cells, while mutations in the Lab initiation site do not affect the production of progeny viruses [33]. The precise deletion of the Lb from the A12 strain of FMDV (serotype A) produced viable viruses in BHK cells, while the mutant virus showed a reduced growth rate and produced smaller plaques [15]. A recent report shows that FMDVs (serotype O) lacking complete Lb coding sequences can be obtained in BHK cells by modifying Lab start codons, while the precise deletion of the Lb coding region alone prevents FMDV replication in primary bovine thyroid cells [19]. In addition, the deletion of the “spacer” region between two initiation codons is not lethal for the virus. These findings imply that the L^pro^ sequence is physiologically associated with FMDV propagation.

Apart from Lab and Lb, another form of L^pro^ has been observed, which is termed sLb^pro^ or Lb’ [34, 35]. sLb^pro^ is generated by the removal of six or seven residues from the C-terminal extension (CTE) of L^pro^ during FMDV infection [36]. The trimming of the CTE of L^pro^ results in different characteristics of sLb^pro^. sLb^pro^ cannot form homodimers like Lb via interactions of the CTE of one monomer with the substrate-binding site of the neighboring one, and vice versa [34, 35]. The Lb homodimers have been observed by X-ray crystallography and nuclear magnetic resonance (NMR) [34, 35], providing weak evidence for intermolecular reactions during self-cleavage. The X-ray structures of the L protease were obtained with the two forms of the protein, Lb (not Lab) and sLb, which additionally were modified (C51A). However, both the kinetic evidence of cleavage efficiencies and the structural evidence provided by NMR study on the monomeric variant of Lb, have strongly indicated an intramolecular mechanism of self-processing. Moreover, the obvious formation of a homodimer suggests that it may have a potential function in the modulation of enzyme activity; the dimer may be a physiologically active form responsible for the cleavage activities after the self-processing [35, 37]. The loss of the last six or seven residues in the CTE does not affect the cleavage efficiencies of sLb^pro^ on the eIF4G site. This is because both Lb and sLb^pro^ use residue C133 and two conserved amino acid residues (D184 and E186) of CTE, mediating binding and cleavage of eIF4GI. However, the cleavage efficiencies of Lb and sLb^pro^ are different during the intramolecular incision of the polyprotein substrate due to the lack of an intact CTE in sLb^pro^, as the presence of at least one intact CTE is more favorable for intermolecular cleavage [38]. Although, the exact role of sLb^pro^ remains unknown, it is thought to have a function during FMDV infection [38]. A putative SAP domain identified in L^pro^ is also involved in the biological activities and functions of L^pro^. The mutation in some sites of the SAP domain lead to the production of different forms of L^pro^; all with varying functions [39].

## Cleavage activity of L^pro^

**Figure 2:**
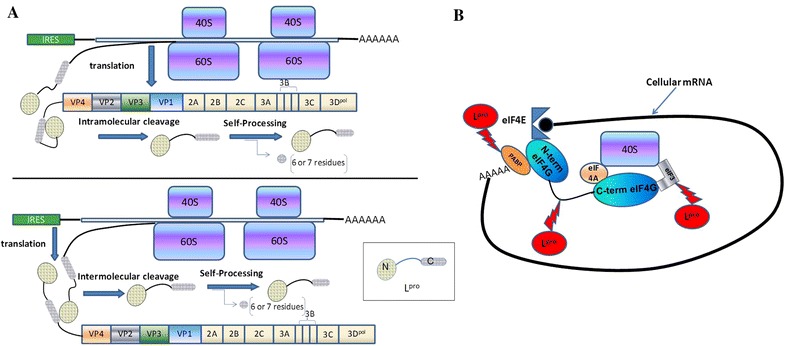
**The cleavage activity of L** ^**pro**^. **A** The self-cleavage activity of L^pro^. L^pro^ can cleave itself from the viral polyprotein translated from the FMDV genome by either an intramolecular or intermolecular reaction. sLb^pro^ is generated by removing six or seven residues from the C terminus of L^pro^ and **B** schematic representation of eIF4G and PABP cleavage induced by L^pro^. L^pro^ can cleave eIF4G, eIF3, and PABP.

L^pro^, the first matured protein of FMDV, self-cleaves from the viral genome ORF-encoding polyprotein. The self-release of L^pro^ is thought to result from both intramolecular [28] and intermolecular [4] cleavage. The sequences of KVQRKLK*GAGQSS at the junction between L^pro^ and viral structural protein precursor (P1-2A) are thought to be the cleavage sites [4] (Figure 2A). In addition to the self-cleavage activity of L^pro^, it can cleave the homologues of host eIF4G in vitro (Figure 2B). The amino acid sequence recognized as the cleavage site of eIF4GI is PSFANLG*RTTLST [40], and VPLLNVG*SRRSQP for eIF4GII [21]. However, there remain some controversies about the precise cleavage sites within eIF4GI and eIF4GII generated by the Lb^pro^, because the cleavage sites of eIF4GI or eIF4GII in the virus-infected cells have not been identified.

L^pro^ is a papain-like cysteine proteinase. Although sequencing shows that L^pro^ shares low nucleotide identity with papain family members [24], the typically conserved catalytic cysteine and histidine residues belonging to papain-like proteinase have been identified in L^pro^ [41]. The catalytic cysteine site is located at the top of the central α-helix, and the catalytic histidine site lies opposite to it on a turn between two β-sheets in the right-hand domain [42]. The most conserved region between papain-like proteases and Lb structures surrounds the active center, particularly the secondary components, α1 and β5–β6 [42].

The crystal structure of L^pro^ (indicating the Lb^pro^) includes a globular domain similar to other members of the papain superfamily cysteine proteinase, and a flexible CTE. L^pro^ also possesses the same overall folding, which resembles the cellular prototype of papain. However, the pro-peptide binding loop and many other loops found in papain are not observed in L^pro^ [42]. Members of the papain proteinase superfamily have a corresponding activity unit, which comprises the catalytic triad of Cys/His/Asn [43]. This catalytic unit of Cys/His/Asp is also present in L^pro^. According to a detailed comparison of the two active sites, certain hydrogen bonds and water molecules localized at the catalytic site are remarkably conserved. Hydrogen bonds stabilize the side-chain amide group contributing to the oxyanion hole in both enzymes. One of the carboxylate oxygen atoms of Asp164 and amide nitrogen atoms of Asn46 form a hydrogen bond in Lb. In papain, the hydrogen bond comprises a P-Ser176 hydroxyl group and P-Gln19 amide oxygen atom. The multiple discrepancies between the structures of L^pro^ and cysteine protease give rise to physicochemical differences between the two enzymes. For example, in the soluble state, when the concentration of cations increases, cysteine protease displays excellent tolerance and keeps its original state, whereas the activity of L^pro^ changes markedly. The fluctuation of pH can significantly influence the activity of L^pro^ because its cleavage activity varies greatly in different pH ranges [34].

## Cleavage of host proteins induced by L^pro^

Eukaryotic cellular translation initiation factor 4F (eIF4F) is a protein complex that recruits ribosomes to bind to host mRNA, initiating cap-dependent translation. This recruitment process is a rate-limiting step and therefore regulates translation [44]. The eIF4F complex comprises eIF4E small cap-binding protein, eIF4G scaffolding protein, and eIF4A ATP-dependent RNA helicase with capped-mRNA. The cap binding factor eIF4E, can bind to a segment of eIF4G to facilitate the formation of the eIF4E/cap-mRNA complex. As a core apparatus of eIF4F complex, eIF4G is a scaffolding protein that provides the binding regions for eIF4E, eIF4A, and RNA elements to form the eI4F complex. The eIF4G protein also provides binding sites that recruit the small ribosomal subunit interacting protein eIF3 (recruiting the 40S ribosomal subunits to the 5′-end of the mRNA in eIF4F complex), poly(A)-binding protein (PABP), and eIF4E kinases Mnk1 (mitogen-activated protein kinase signal-integrating kinase1) and Mnk2, regulating host mRNA translation [45].

eIF4G proteins possess two homologous proteins in yeast, eIF4GI (*TIF4631*) and eIF4GII (*TIF4632*), sharing a similar function. Both of them contain the conserved binding sites for eIF4E, PABP, eIF3 and RNA. For eIF4GI, it is reported that its N-terminal portion provides the binding sites for eIF4E and PABP, whereas eIF4A and eIF3 bind to the C-terminal portion of eIF4GI [46, 47]. Some picornaviruses including poliovirus, human rhinovirus 2, and FMDV can effectively cleave the eIF4GI, yielding N- and C-terminal fragments [40, 47]. FMDV Lb protease can also cleave eIF4GII, generating a C-terminal fragment [48]. The loss of integrity of eIF4GI and eIF4GII blocks the formation of the eIF4F complexes, which directly influences the cellular cap-dependent translation. However, the C-terminal fragment of both eIF4G proteins containing the binding sites for eIF4A and eIF3 can still bind to the FMDV IRES as efficiently as the non-processing eIF4GI and eIF4GII respectively [47, 48]. Studies over the last two decades have shown that regulation of host and viral mRNAs by eIF4G is achieved by different mechanisms. Viral protein synthesis initiated at two distinct sites from artificial fusion genes is independent of the cap-binding eIF4F complex in the presence of IRES [29]. Furthermore, the cleavage products of eIF4GI (C-terminal portion) stimulate the translation of uncapped RNAs and those carrying IRESs [49]. The interaction of the two eIF4G proteins with IRES is an essential event for promoting IRES activity. Therefore, viral RNA translation is unaffected [48, 50].

eIF4GI is a major form of eIF4G, which correlates with inhibition of cellular cap-dependent protein synthesis within FMDV-infected cells [4, 40]. However, cellular protein synthesis can still be maintained at a reduced level, with the complete loss of intact eIF4GI when virus replication is inhibited [51]. The discovery of human eIF4GII, which appears functionally analogous to eIF4GI, has resolved this puzzle [52]. The shut-off of host cell protein synthesis significantly decreases the expression of various cytokines and the major histocompatibility complex (MHC), resulting in delayed host antiviral effects. However, viral uncapped RNA can be translated through an IRES that is independent of intact eIF4G [53]. Therefore, the virus quickly takes over the host machinery to propagate vast numbers of progeny. FMDV lacking L^pro^ is unable to escape the antiviral response and is not disseminated in the infected animals [16].

Apart from the cleavage of eIF4G, L^pro^ can cleave a series of cellular proteins, such as eIF3a, polypyrimidine tract-binding protein (PTB), PABP and Gemin5, which are involved in the control of translation, and death domain associated protein (Daxx), a key factor that crosslinks the apoptosis, innate immune responses and transcription control, to interfere with various cellular pathways during viral infection [54]. The events associated with the extent of cytopathic effects in FMDV-infected cells are proteolysis of PTB, which is involved in mRNA stability and RNA localization, interaction of PABP with the entire FMDV 3′-UTR, and the binding of two subunits of eIF3 (eIF3a and b) with the IRES [11]. Recently, Piñeiro et al. [54] reported that the RNA-binding protein Gemin5 is also a target of L^pro^. Gemin5 is the RNA-binding factor of a large macromolecule of the survival of motor neuron (SMN) complex, which acts as a down-regulator of cellular mRNA translation and IRES-driven translation initiation [55]. L^pro^ recognizes the sequence RKAR of Gemin5 and induce its proteolysis, yielding two stable products of molecular weight 85 and 57 kDa within FMDV-infected cells [54]. Daxx has also been identified as a substrate of L^pro^, and the RRLR motif is the recognition site. Daxx is a ligand of Fas, acting as a multifunctional adaptor protein in the process of apoptosis, innate immune responses, and in transcriptional regulation [56]. The cleavage recognition site for L^pro^ in PABP1 has not been identified experimentally. The sequence similarity with other L^pro^ substrates and the molecular weight of the proteolysis product imply this characteristic [11], and it is deduced that a novel motif containing sequence (R)(R/K)(L/A)(R) is a putative target sequence of L^pro^. Hence, neuroguidin, an eIF4E and cytoplasmic polyadenylation element binding protein (CPEB) that plays an important role in neuronal development [57], is hypothesized to be a potential target of L^pro^, with the target sequence as AKRRALS [54]. Furthermore, eIF3a and b are essential to the assembly of the translation initiation complex, and are associated with PABP and RNA-binding protein PTB. This is involved in mRNA stability and RNA localization and can be proteolysed by FMDV L^pro^, whereas PABP can be partial cleaved by L^pro^ [11]. All these studies suggest that L^pro^ can cleave various host proteins and has potential multifunctional roles.

Other than these identified substrates of L^pro^, various IRES-binding factors that are targets of other picornavirus proteases may contribute to understanding the link between these proteins and L^pro^. These factors include poly(rC)-binding protein 2, Gemin3 (RNA helicase that is a component of the SMN complex), RIG-I (retinoic acid-inducible gene 1; a cytoplasmic RNA helicase that senses viral infection), MAVS (mitochondrial antiviral-signaling protein), TRIF (Toll/interleukin (IL)-1 receptor domain-containing adaptor inducing IFN-β or innate immune adaptor molecules), and the stress granules protein G3BP [58–62].

## Suppression of IFN production mediated by L^pro^

FMDV infection triggers the activation of various pattern recognition receptors (PRRs) and induces a series of antiviral responses; with the transcription factor NF-κB acting as a sensor in response to the general alteration of the cellular environment. After the PRRs recognize the pathogens, the coordinated activation of various transcription factors including NF-κB, IFN regulatory factor (IRF)3 and IRF7, are initiated to induce early expression of type I IFNs and activate host antiviral responses [63].

PRR-induced signal transduction can activate NF-κB to translocate into the nucleus through degradation of NF-κB inhibitor. Nuclear translocation of NF-κB is followed by its binding to the promoter sequences of many genes to initiate their transcription. The expression of various cytokine genes such as the proinflammatory factors, chemokines, and adherence factors is greatly enhanced to induce antiviral responses [64, 65]. NF-κB also promotes secretion of IFN-α/β and their binding to corresponding receptors. This activates the JAK/STAT signaling pathway, which subsequently induces the expression of hundreds of IFN-α/β-stimulated genes (ISGs). ISGs are a class of antiviral genes that directly encode antiviral proteins that suppress virus propagation at different stages of the viral replication cycle [66]. It was recently reported that the enhanced expression of ISGs increases antiviral effects on FMDV [67]. IRFs are transcription factors that are pivotal for inducing activation of IFN-α/β during virus infection; IRF3 and IRF7 are crucial for virus-triggered IFN-α/β secretion [68]. IFN-α/β belong to the family of type I IFNs and serve as the first line of host defenses, displaying critical antiviral activity [69]. In addition, IFN-λ, a type III IFN, possesses IFN-like activity and is suggested to be a potent antiviral factor that is effective against many viruses [70, 71].

FMDV L^pro^ acts as an antagonist of innate immune responses mainly eliciting the IFN-α/β specific antiviral activity at both protein and mRNA levels. The down-regulation of IFN expression at least in part corresponds to the cleavage of eIF4G by L^pro^. Both genetically engineered FMDV lacking L^pro^ (A12-LLV2) and wild-type FMDV (A12-IC) were observed to induce the production of IFN-α/β mRNAs in secondary cells from susceptible animals. However, the A12-LLV2 mutant induces greater antiviral activity than the wild type as a consequence of failing to shut off the expression of host cell protein, including IFN-α/β [18]. L^pro^, blocks IFN protein synthesis, as well as synthesis of IFN-β mRNA and at least three ISGs mRNAs [10], including double-stranded RNA-dependent protein kinase (PKR) which plays an important role in inhibition of FDMV replication, 2′, 5′ oligoadenylate synthetase 1 (OAS1) and myxovirus resistance protein 1 (Mx1). Using microarray technology, a transcriptional profile associated with the antiviral responses against FMDV was systematically analyzed. The results suggested that L^pro^ significantly inhibits NF-κB-dependent gene expression including expression of IFN-β and ISGs during FMDV infection [72]. Furthermore, it was found that during the acute infection phase, levels of type I IFN in the serum from infected animals significantly increased [73]. These studies indicate that type I IFN production is associated with antiviral effects against FMDV infection and is important in antiviral immune regulation. L^pro^ as a critical virulence factor of FMDV is capable of using multiple strategies to suppress the production of IFNs.

Many picornaviruses have evolutionarily developed subtle strategies that target host factors to subvert IFNs signaling pathways, and survive and replicate in host cells. For example, enterovirus 2A^pro^ counteracts IFNs responses in infected cells by cleaving melanoma differentiation-associated protein 5 (MDA5) and MAVs [74], while the mengovirus utilizes L^pro^ to prevent the production of IFN-α/β by inactivating iron/ferritin-mediated activation of NF-κB [75]. Cardiovirus L^pro^ induces cellular nuclear transport inhibition by binding to a key trafficking regulator RanGTPase [76].

Accumulating evidence shows that L^pro^ of FMDV inhibits IFN production through interfering with the IFN signaling pathways. De Los Santos et al. determined that L^pro^ can restrict the induction of IFN-β mRNA [10]. The restriction is partially built on the control of transcription factors and their upstream signaling factors by L^pro^. L^pro^ was shown to be associated with the downregulation of nuclear p65/RelA during FMDV infection [8]. P65/RelA is the core component of NF-κB, and a decrease in the integrity of p65/RelA may lead to the reduction of NF-κB. This ultimately results in downregulation of IFN-β expression and attenuation of host innate immune responses [8]. The mechanism involved in the downregulation of p65/RelA induced by L^pro^ remains unclear. Whether the disappearance of p65/RelA is mediated by the cleavage activity of L^pro^ has not been confirmed, since no cleavage products of p65/RelA have been determined and no cleavage sites have been mapped until now. Wang et al. observed that L^pro^ decreases IRF-3-induced IFN-α/β expression by reducing IRF-3 and IRF-7 expression [77]. L^pro^ can also suppress the secretion of IFN-λ1 by disrupting the IRFs and NF-κB activation, which is crucial for IFN-λ1 expression [23]. The strategy adopted by L^pro^ is to cut off the connection between the IFN promoters and transcription factors by decreasing the number of transcription factors, thereby inactivating IFN transcription. L^pro^ can also use its deubiquitination activity to prevent IFN-α/β production by reducing ubiquitination of several type I IFN signaling molecules (details in next section). All these results indicate that L^pro^ uses various strategies to suppress IFN-α/β production and promote FMDV replication.

## Deubiquitination activity of L^pro^

It is well known that the activation of many signaling events that connect the sensors with the transcription factors are regulated by ubiquitination enzymes. The conjunction of ubiquitin with the signaling molecules contributes to the activation of several of these signaling events [66, 78]. However, there are also deubiquitinating enzymes (DUBs) [79] that can inactivate this complex by cleaving ubiquitin from its substrate proteins [80]. DUBs belong to the proteinase superfamily, of which 100 members have been identified in humans. DUBs can be classified into two main categories, metalloproteases and cysteine proteases [79]. The DUBs such as, A20, cylindromatosis (CYLD) protein, and deubiquitinating enzyme A (DUBA) negatively regulate the ubiquitination process, and hence, are key regulators in antiviral responses. For example, A20 is involved in downregulation of NF-κB activation, negatively regulating host antiviral responses. A20 is a DUB that can remove K63-linked ubiquitin from the ubiquitinated receptor-interacting protein (RIP) [80]. RIP is a serine/threonine kinase that contains a death domain which can interact with the death receptors Fas and tumor necrosis factor (TNF) receptor 1 to mediate activation of NF-κB [81]. Deubiquitination of RIP directly abates activation of the NF-κB signaling pathway [80]. Yokota et al. recently reported that measles virus P protein upregulates A20 to repress Toll-like receptors, inhibiting activation of NF-κB [82].

Bioinformatics analysis suggests that Lb has a potential DUB structure and conserved DUB catalytic residues (Cys51 and His148). The observed catalytic residues are highly conserved in the Lb of all seven serotypes of FMDV. Structural analysis indicates that Lb possesses a topology similar to DUB ubiquitin-specific 14 and resembles papain-like protease (PL^pro^) of severe acute respiratory syndrome coronavirus (SARS-CoV) [22, 83]. It has been observed that mutation of the SAP box (I83A/L86A) or the catalytically active site (C51A or D163 N/D164 N) of Lb results in the inactivation of DUB activity of L^pro^ [22].

## L^pro^ counteracts innate immune responses through its DUB activity

Over the course of long-term evolutionary processes, many viruses have developed sophisticated strategies to antagonize host antiviral responses. Redirecting the cellular ubiquitination system to suppress innate antiviral immune signaling pathways is one of the strategies. For example, rotavirus NSP1 blocks NF-κB- and IRF- dependent transcription of type I IFN by inducing proteasome-mediated degradation of IRF3/5/7 or inhibiting IκB-α (inhibitor of NF-κB) degradation to prevent NF-κB activation [84, 85]. The accessory proteins, Viral Protein R and Virion Infectivity Factor of HIV can independently hijack the cellular ubiquitination system to decrease IRF-3 expression through proteasomal degradation and promote virus replication [86]. As a result, the production of host antiviral ISGs and proinflammatory factors is reduced and the antiviral innate responses are attenuated. Moreover, many viruses can hijack host ubiquitination systems to facilitate viral evasion, genomic replication, and exocytosis [87].

In addition to hijacking the host ubiquitination system for virus replication, many viruses have also developed the ability to disrupt cellular ubiquitination machinery to terminate or block several signaling transduction pathways responsible for the induction of antiviral responses [88]. So far, the PL^pro^ of several coronaviruses such as, porcine epidemic diarrhea virus, SARS-CoV, and Middle East respiratory syndrome (MERS-CoV) have been shown to possess deubiquitination activity that antagonizes IFN production, indicating that PL^pro^ is a multifunctional protein [89, 90]. Similarly, FMDV L^pro^ is a papain-like protease that acts as an antagonist of IFN by negatively regulating IFN transcription and IFN mRNA translation [8, 18, 42, 77].

**Figure 3:**
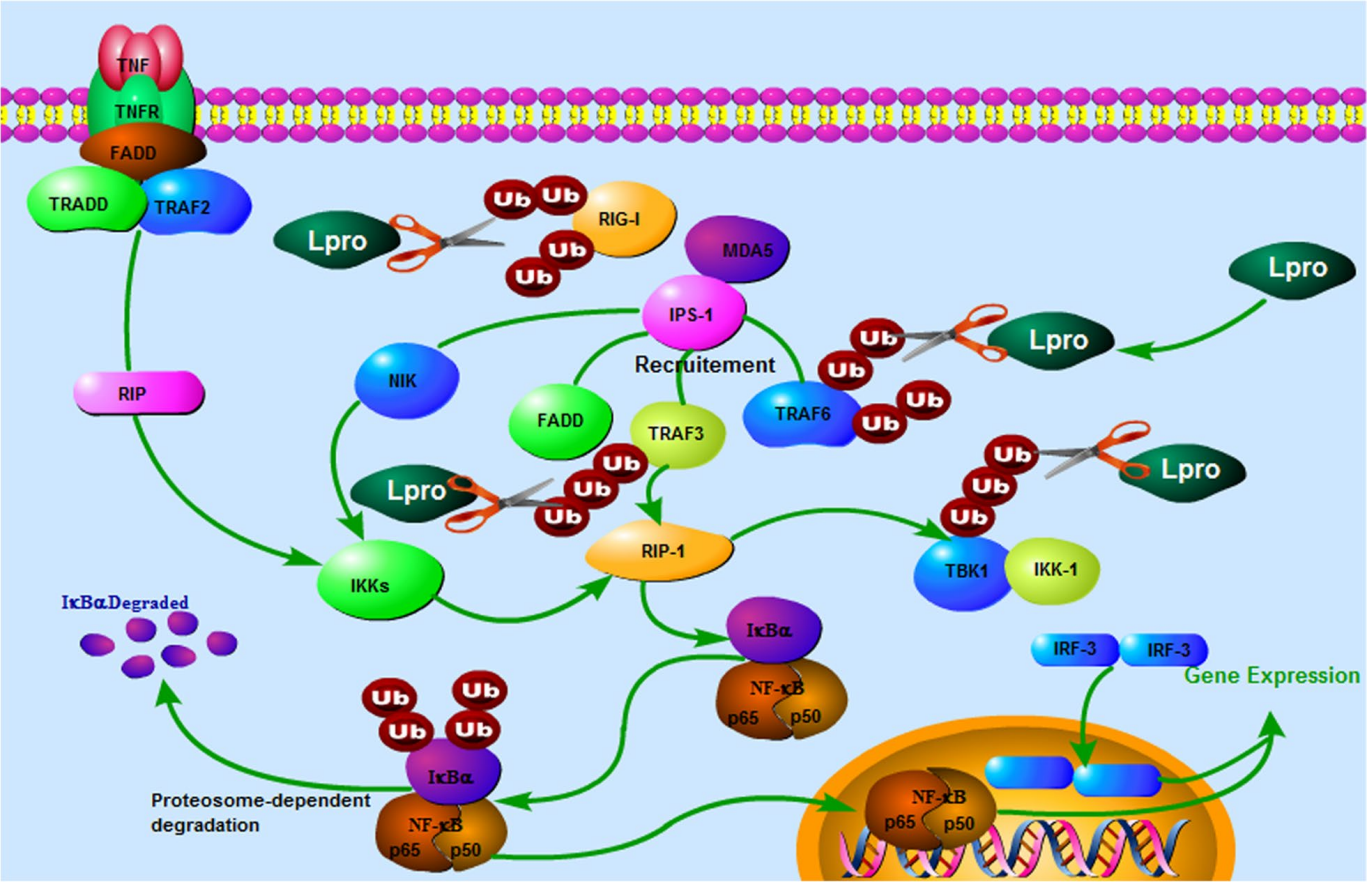
**DUB activity of L** ^**pro**^ **in innate immune signaling pathways.** L^pro^ can deubiquitinate several adaptor proteins including RIG-I, TRAF3, TRAF6, and TBK1. Deubiquitination of these proteins contributes to the attenuation of host innate immune responses.

A recent study from Wang et al. has identified a DUB-like activity of Lb of FMDV [22]. It was observed that Lb significantly inhibited ubiquitination of several adaptor signaling molecules of type I IFN pathway, including RIG-I, TBK1, TRAF3, and TRAF6 (Figure 3). The results of sequence alignment and structural bioinformatics analyses indicate that L^pro^ and ubiquitin-specific protease (USP)14 share similar topology [91]. The DUB activity of Lb was further confirmed through observation of the inhibitory effects of Lb on ubiquitination of RIG-I, TRAF3, TRAF6, and TBK1, which eventually prevents activation of the type I IFN pathway. This DUB activity can be abrogated through mutation of the conserved catalytic sites of Lb. The deubiquitinating processes mediated by Lb are similar to those mediated by DUBA and CYLD. Future studies should focus on whether the DUB activity of Lb is involved in the signaling pathways regulated by A20.

## A putative SAP domain identified in L^pro^

De Los Santos et al. discovered that FMDV L^pro^ contains a putative SAP domain (scaffold-attachment factor (SAF)A and SAFB, apoptotic chromatin-condensation inducer in the nucleus (ACINUS), and protein inhibitor of activated STAT (PIAS) domain) [39]. SAP is a conserved domain which usually exists in the eukaryotic proteins and involved in nucleic acid binding, DNA metabolism, DNA repair, chromosomal organization, apoptosis, transcriptional regulation, and immune regulation [92].

SMART software analysis of FMDV L^pro^ predicted an SAP domain between amino acids 47 and 83 of Lb. This putative SAP domain in L^pro^ shows >80% amino acid homology with other SAP domains of eukaryotic proteins. Three-dimensional analysis indicates that L^pro^ and the eukaryotic cellular SAP domains share almost the same α-helix-turn-α-helix structure, in which only two amino acid insertions found in the two α-helices of L^pro^ differed from other cellular SAP domains [39]. Furthermore, a motif of IQKL sequence in L^pro^ resembles the LXXLL signature motif that is mostly found in the SAP domain of PIAS. All these observations demonstrate the presence of a putative SAP domain in L^pro^.

The eukaryotic SAP domain is usually implicated in PIAS-associated functions. The SAP motif in PIAS has been conserved in evolution, from yeast to humans, and this functional motif can recognize and bind to the AT-rich sequence of scaffold/matrix attachment regions (S/MARs) of eukaryotic chromosomes. S/MAR is usually located close to the enhancer sequence so that it provides a special microenvironment for transcription [93]. PIAS is a negative regulator in host antiviral immunity. For instance, *pias* gene knockout mice show more resistance to bacterial infection and improved antiviral responses to vesicular stomatitis virus. It is proposed that PIAS affects the expression of >60 genes, most of which are cytokine-induced and pathogen-activated genes involved in NF-κB and STAT signaling pathways. PIAS1 and PIASy are key proteins of the PIAS family and act as inhibitors to negatively regulate NF-κB- and STAT-dependent gene expression [94]. Furthermore, PIASy adopts distinctive mechanisms to inhibit virus-induced and IFN-stimulated transcription [95]. Intriguingly, some viral proteins are localized in the S/MAR regions, suggesting an interaction between viral proteins and that S/MAR may block host antiviral activities [96]. In addition, there is evidence showing that the VP35 protein of Ebola virus utilizes PIAS to promote sumoylation of IRF7, thus contributing to inhibition of IFN production in immune cells [97]. Until now, whether L^pro^ can adopt an analogous way of using PIAS in inhibiting cellular antiviral activities remains unclear. However, the N-terminal portion of PIAS3 containing the SAP domain was verified to block the NF-κB activation through binding to the p65/RelA subunit of NF-κB [98], whether L^pro^ can use this manner to interrupt activation of NF-κB remains unclear.

## The SAP domain is important for L^pro^ activity

Zhu et al. found that expression of various IFN-inducible genes, chemokines or transcription factors, especially NF-κB-dependent gene expression in L^pro^ SAP domain mutant FMDV-infected bovine cells was significantly enhanced compared with the wild-type FMDV-infected cells [72]. De los Santos and his co-workers revealed that SAP domain is a determinant for L^pro^ nuclear subcellular localization. In FMDV-infected cells, L^pro^ progressively translocates to the nucleus, whereas mutation of two residues at positions 55 and 58 of L^pro^ (SAP mutant) significantly prevents nuclear translocation of L^pro^ without affecting the cleavage of eIF4G. This suggests that the SAP domain affects retention of L^pro^ in the nucleus within the FMDV-infected cells. The proper subcellular localization of L^pro^ in the nucleus is deemed to mediate the L^pro^-dependent degradation of p65/RelA. Observations concerning SAP-related cellular antiviral responses suggest that in SAP-mutant FMDV-infected cells, the mRNA expression levels of several NF-κB-dependent cytokines, chemokines, and ISGs are higher than in wild-type FMDV-infected cells [39]. Collectively, the aforementioned results demonstrate that subcellular localization of L^pro^ in the nucleus is an important factor in the suppression of innate immune responses, and that the SAP domain is involved in this process. Besides, a recent study demonstrated that the catalytic activity and SAP domain of L^pro^ were required for suppressing poly(I:C)-induced IFN-λ1 production [23].

Diaz-San Segundo et al. found that inoculation of pigs with SAP-mutant FMDV (I55A and L58A mutations were introduced in L^pro^) can induce early protection against FMD [99]. No clinical signs of FMD, viremia, or virus shedding were observed, even when the pigs were inoculated at 100-fold higher doses than those required to cause clinical signs with wild-type FMDV. The SAP-mutant FMDV elicited strong adaptive immune responses that provided complete protection against wild-type FMDV infection. Impressively, the neutralizing antibody response was induced as early as 2 days post-inoculation and lasted for at least 21 days after inoculation. In the blood of pigs inoculated with SAP mutant virus, expression of IFN-α, TNF-α, IL-1, and IL-6 was higher than in pigs inoculated with the wild-type virus. Zhu et al. reported that FMDV manipulates ubiquitin-activating enzyme one to promote viral replication, and the SAP domain of L^pro^ was involved in this process, which indicates that SAP maybe has a novel role [100]. All these studies suggest that FMDV L^pro^ plays an important role in virus replication process, and the SAP domain may be a critical region for the maintenance of the biological activities of L^pro^.

## Conclusions

FMDV has evolved numerous strategies to evade host antiviral responses. In order to survive and replicate in host cells, the virus has developed various ways to impair or suppress the induction and activation of antiviral responses, utilizing viral nonstructural proteins. L^pro^ and 3C^pro^ are the main viral factors that antagonize host immune responses, with L^pro^ being one of the most well-characterized proteins. L^pro^ can cleave numerous host proteins, inhibit cellular protein expression, and deubiquitinate some crucial molecules that are essential for the activation of antiviral pathways and signal transduction. Intensive study of FMDV L^pro^ has uncovered several mechanisms by which FMDV replicates in host cells and suppresses host antiviral responses utilizing L^pro^ (Table 1). However, these observations represent only the “tip of the iceberg” and several questions regarding the different forms of L^pro^ and the pathways involved in L^pro^-mediated antagonistic effects need to be answered. Further studies are necessary to elucidate these unanswered questions and the multifunctional role of L^pro^ in FMDV infection.

**Table 1:** The target proteins and the multifunctional role of L^pro^.

Biological functions of L^pro^	Auto cleavage	Cleavage activities			Deubiquitination activity	Unknown
Target proteins	Viral polyprotein [4]	eIF4GI [40]; eIF4GII [21]	Gemin5 [54] eIF3a and b; PTB;PABP1 [11]	Daxx [54]	RIG-I,TBK1,TRAF3 and TRAF6 [22]	NF-κB [8]; IRF-3/7 [77]
Recognition site/region	KVQRKLK^201^*GAGQSS	eIF4GI:PSFANLG^674^ *RTTLST; eIF4GII:VPLLNVG^700^ *SRRSQP	PABP: putative motif RRSL; Gemin5:IKKRKAR^846^*SLLPLS(RKAR motif); others: unknown	Daxx:VLARRLR^360^*ENRSLA (RRLR motif)	The polyubiquitination chain of ubiquitinated signaling molecules	Unknown
The effects induced by L^pro^	Directing the release of L^pro^ from the nascent polyprotein	Shutting off cellular translation	Control of cellular translation	Regulation of apoptosis and innate immune antiviral responses	Impairing innate immune signaling pathways	Restricting antiviral activities of IFN-α/β and IFN-λ1

## Abbreviations

FMD: foot-and-mouth disease
FMDV: foot-and-mouth disease virus
L^pro^: leader protein
5′-UTR: 5′ untranslated region
3′-UTR: 3′ untranslated region
ORF: the open reading frame
eIF4G: eukaryotic translation initiation factor 4 gamma
NF-κB: nuclear factor kappa B
NEMO: NF-κB essential modulator
KPNA1: karyopherin α1
IFN: interferon
DUB: deubiquitination
NMR: nuclear magnetic resonance
eIF4F: eukaryotic cellular translation initiation factor 4F
PABP: poly(A)-binding protein
Mnk: mitogen-activated protein kinase signal-integrating kinase1
IRES: internal ribosome entry site
MHC: major histocompatibility complex
PTB: polypyrimidine tract-binding protein
Daxx: death-domain associated protein
CPEB: cytoplasmic polyadenylation element binding protein
PCBP: poly(rC)-binding protein
RIG-I: retinoic acid-inducible gene 1
MAVS: mitochondrial antiviral-signaling protein
TRIF: Toll/interleukin (IL)-1 receptor domain-containing adaptor inducing interferon-β or innate immune adaptor molecules
PRRs: pattern recognition receptors
IRF: IFN regulatory factor 3
OAS1: 2′, 5′ oligoadenylate synthetase 1
RIP: receptor interacting protein
TRAF: TNF receptor-associated factor
TBK1: TANK binding kinase 1
PL^pro^: papain-like protease
PEDV: porcine epidemic diarrhea virus
SARS-CoV: severe acute respiratory syndrome coronavirus
MERS-CoV: middle East respiratory syndrome
SAP domain: scaffold-attachment factor (SAF)A and SAFB, apoptotic chromatin-condensation inducer in the nucleus (ACINUS), and protein inhibitor of activated STAT (PIAS) domain
S/MARs: AT-rich sequence of scaffold/matrix attachment regions
TNF: tumor necrosis factor
